# Identification of Potential Ferroptosis Key Genes in the Pathogenesis of Lumbosacral Spinal Root Avulsion by RNA Sequencing and Bioinformatics Analysis

**DOI:** 10.3389/fmolb.2022.902607

**Published:** 2022-08-05

**Authors:** Zhibin Zhou, Jiajia Lu, Jun Ma, Lei Zhu

**Affiliations:** ^1^ Department of Orthopaedics, General Hospital of Northern Theater Command, Shenyang, China; ^2^ Department of Orthopaedics, Second Affiliated Hospital of Naval Medical University, Shanghai, China; ^3^ Naval Medical Center of PLA, Naval Medical University, Shanghai, China

**Keywords:** ferroptosis, LSRA, RNA sequencing, bioinformatics, neuronal death

## Abstract

**Objective:** Ferroptosis is a type of cell death involved in various human diseases, including nerve injury. However, the role of ferroptosis in lumbosacral spinal root avulsion (LSRA) remains unknown. This study aims to investigate whether ferroptosis is induced after LSRA and the key ferroptosis-related genes and their potential function in LSRA.

**Methods:** The biochemical and morphological changes of ferroptosis were determined by detection of iron accumulation and by transmission electron microscopy in a rat LSRA model. The transcriptional expression profile following LSRA was investigated by RNA sequencing and ferroptosis-related genes were downloaded from FerrDb and used to identify ferroptosis differentially expressed genes (DEGs). The differential expressions of ferroptosis DEGs were confirmed by qRT-PCR analysis. The potential functions of ferroptosis DEGs were revealed by DAVID 6.8 and WebGestalt. A protein–protein interaction (PPI) network and gene–miRNA interaction network were further constructed to identify key modules in ferroptosis DEGs, and the results were verified by qRT-PCR and western blot analysis.

**Results:** LSRA was followed by ferroptosis-specific changes, such as shrunken mitochondria and increased iron accumulation, that can be alleviated by ferroptosis inhibitor deferoxamine (DFO). A total of 2,446 DEGs and 46 ferroptosis DEGs were identified after LSRA, and over 90% of the ferroptosis DEGs were confirmed to be differentially expressed following LSRA, which can also be eliminated by DFO treatment. Functional analysis demonstrated significant enrichment of the ferroptosis DEGs in pathways related to the oxidative stress response, the HIF-1 signaling pathway, and the tumor necrosis factor signaling pathway. PPI network analysis demonstrated that a set of key modules in ferroptosis DEGs were related to the HIF-1 signaling pathway: Il6, Nos2, Stat3, Hif1a, Vegfa, Cdkn1a, and Rela. Construction of a gene–miRNA network predicted miRNAs targeting four key ferroptosis DEGs—Stat3, Hif1a, Vegfa, and Rela, and further western blot analysis confirmed their upregulation after LSRA, which can be alleviated by DFO pretreatment.

**Conclusion:** The data revealed the induction of ferroptosis in a rat LSRA model and identified possible regulatory roles for ferroptosis-related genes in the molecular mechanisms of LSRA, which provides new insights into the pathogenesis and helps to find new molecular targets for the treatment of LSRA.

## Introduction

Lumbosacral spinal root avulsion (LSRA), commonly caused by traffic accidents or high fallings, occurs when the lumbosacral nerve roots are torn off from the spinal cord (Chin and Chew, 1997). Due to the widespread death of neurons in the corresponding spinal segment, LSRA may result in devastating dysneuria in the lower extremities, such as motor dysfunction, neuropathic pain, and numbness (Ohlsson et al., 2013). Although increasing efforts have been dedicated to developing therapeutics for LSRA, and several novel surgical strategies have been proven to be effective, the clinical prognosis remains poor (Gu et al., 2004; Zhu et al., 2015). Therefore, exploring novel medical approaches for the management of LSRA is vitally important.

The death of neurons has been regarded as a key factor leading to unsatisfactory functional recovery after LSRA, and we previously found that two canonical cell death pathways, apoptosis and autophagy, were induced in neurons of the corresponding spinal cord and were closely related to the neuronal survival rate (Jiang et al., 2014; Zhu et al., 2016). Ferroptosis, characterized by the requirement for iron and the accumulation of reactive oxygen species (ROS), is a type of cell death different from apoptosis or autophagy (Dixon et al., 2012). Numerous studies have shown that ferroptosis is closely related to the occurrence and development of various human diseases. Early research on ferroptosis mainly focused on cancers (Dolma et al., 2003; Yang and Stockwell, 2008), while further studies revealed ferroptosis in many other diseases, such as acute renal failure (Müller et al., 2017), ischemia-reperfusion injury (Yamada et al., 2020) and Parkinson’s disease (Ayton et al., 2013). In recent years, ferroptosis has also attracted attention in acute nervous system injuries, including traumatic brain injury (Kenny et al., 2019) and spinal cord injury (Zhang et al., 2019). However, the relationship between ferroptosis and LSRA remains to be explored.

Here, to investigate whether ferroptosis is induced after LSRA and the key ferroptosis-related genes and their potential function in LSRA, we determined the biochemical and morphological changes associated with ferroptosis in a rat LSRA model and screened the transcriptional expression profile of spinal cord tissue from LSRA and sham-operated rats. Differentially expressed genes (DEGs) were identified and intersected with the ferroptosis dataset to obtain ferroptosis DEGs. The potential functions of ferroptosis DEGs were revealed by integrated bioinformatics analysis, and then the key ferroptosis-related genes were identified and verified. Our results demonstrated the induction of ferroptosis in a rat LSRA model and identified possible regulatory roles for ferroptosis-related genes in LSRA, which might help to reveal the underlying mechanism of ferroptosis in LSRA, thus providing new insights into the pathogenesis of LSRA.

## Methods

### Animals

Adult male Sprague-Dawley rats (200–220 g) aged 8 weeks were obtained from Shanghai SLAC Laboratory Animal Co., Ltd. Rats were housed under a 12-h light/dark cycle and a humidity- and temperature-controlled environment with free access to food and water. All experimental procedures on animals were approved by the Animal Ethics Committee of the Second Affiliated Hospital of Navy Medical University (Shanghai, China) and performed according to their guidelines.

### Establishment of a Rat LSRA Model and Experimental Design

A rat LSRA model was established as described previously (Zhu et al., 2016). LSRA surgery was performed by explosion and avulsion of the right L4–L6 nerve roots in rats anesthetized with an intraperitoneal injection of pentobarbital sodium. For Sham surgery, the right L4–L6 nerve roots were only exposed but not avulsed. Deferoxamine (DFO, Novartis, Basel, Switzerland) treatment was performed as previously reported (Yao et al., 2019). For a short period, DFO was dissolved in 0.9% normal saline and intraperitoneally injected into rats 30 min before surgery, and 0.9% NaCl was used as a control.

A total of 48 rats were randomly assigned to the following groups: for the DFO treatment experiment, the Sham group (*n* = 10), which received Sham surgery; the LSRA group (*n* = 10), which received LSRA surgery and saline treatment (1 ml/kg); and the LSRA + DFO group (*n* = 10), which received LSRA surgery and DFO treatment (100 mg/kg). For the RNA sequencing experiment, the Sham group (*n* = 9) received Sham surgery and the LSRA group (*n* = 9) received LSRA surgery.

### Transmission Electron Microscope

Spinal cord tissues from the Sham, LSRA, and LSRA + DFO groups were harvested 1 day after surgery and fixed in 2% paraformaldehyde fix solution and 2.5% glutaraldehyde in 0.1 M phosphate buffer overnight. Tissues were then cut into small pieces and fixed in 1% osmium tetroxide in 0.1 M phosphate buffer, dehydrated in an ethanol series, and embedded in Epon. Ultrathin sections were obtained and stained with uranyl acetate and lead citrate, and images were examined under a transmission electron microscope (JEM-1200EX, JOEL, Japan).

### Determination of the Iron Concentration

Spinal cord tissues from the Sham, LSRA, and LSRA + DFO groups were harvested and homogenized under ice-cold conditions. The iron levels were determined using an iron assay kit (Colorimetric) (ab83366, Abcam, Cambridge, UK) according to the manufacturer’s guidance. The iron concentration was calculated by comparing the OD value at 520 nm detected using a spectrophotometer to the standard curve.

### RNA Isolation and RNA Sequencing

The right L4–L6 segments of the spinal cord were harvested for RNA isolation 1 day after surgery. Total RNA was extracted and prepared for RNA sequencing. The spinal cord tissues of three rats in the same group were mixed into one sample, so each group had three samples (*n* = 3 rats/sample and *n* = 3 samples/group). High-throughput RNA sequencing was performed by OE Biotech (Shanghai, China). For a brief period, RNA libraries were constructed and purified using a reaction kit, RNA sequencing was performed using the Illumina HiSeq™ 4000 sequencing platform (Illumina, California, United States), and 150 bp paired-end reads were generated.

### Identification of Differentially Expressed Genes and Ferroptosis DEGs

After RNA sequencing, raw data were obtained and mapped to the rat reference genome (Kim et al., 2015). Quantification of mRNA transcript abundance was performed by normalized expression values as fragments per kb per million reads using bowtie2 (Langmead and Salzberg, 2012), and *p* < 0.05 and |fold change| ≥ 2 were used as criteria for the identification of DEGs. Furthermore, ferroptosis DEGs were identified by intersecting these DEGs with 259 genes downloaded from the Ferroptosis Database (FerrDb).

### Functional Enrichment Analysis

To investigate the functions of ferroptosis DEGs, DAVID 6.8 and WebGestalt with different algorithms were used. First, gene ontology (GO) analysis and Kyoto encyclopedia of genes and genomes (KEGG) pathway analysis were performed to explore the biological functions and enriched pathways of ferroptosis DEGs in LSRA. Then, gene set enrichment analysis (GSEA) was conducted by uploading the ferroptosis DEGs to WebGestalt.

### Protein–Protein Interaction Network Analysis

Protein–protein interactions (PPIs) were conducted by uploading the ferroptosis DEGs to STRING (Szklarczyk et al., 2017). The PPI network was obtained by visualizing the genes with interaction scores >0.4 using Cytoscape. To identify key modules in the network, molecular complex detection (MCODE) (Bader and Hogue, 2003) was used for clustering analysis, and the identified clusters were also visualized using the Cytoscape software.

### miRNA Prediction of the Indicated Genes

To reveal the roles of the indicated genes identified by PPI interaction network analysis in spinal root avulsion, miRWalk 2.0 was used to predict miRNAs that target these genes. A gene–miRNA interaction network was built with the predicted miRNAs using Cytoscape. In addition, miRTarBase (https://mirtarbase.cuhk.edu.cn/), a database of accumulated miRNA–target interactions that was validated by experiment, was used for further identification of potential miRNAs, and they were also visualized in the gene–miRNA interaction network.

### qRT-PCR Analysis of Ferroptosis DEGs and Predicted miRNAs

The expression levels of ferroptosis DEGs (the Sham, LSRA, and LSRA + DFO groups) and predicted miRNAs (the Sham and LSRA groups) were determined by qRT-PCR analysis. Total RNA was isolated from the spinal cord tissues of rats from different groups (*n* = 10 for each group). RNA was transcribed into cDNA for mRNA analysis, and a stem-loop qRT-PCR assay was applied for miRNA analysis using an ABI PRISM^®^ 7500 Sequence Detection System. The primers used in this study are shown in Supplementary Table S1. The expression levels of mRNA and miRNA are normalized to those of glyceraldehyde 3-phosphate dehydrogenase (GAPDH) and U6, respectively. Data were analyzed by comparing the 2^−ΔΔCt^ values.

### Western Blotting

Western blot analysis was performed to measure the protein levels of Rela, Stat3, Hif1a, and Vegfa in the Sham, LSRA, and LSRA + DFO groups following standard methods. For a brief period, total protein was extracted using radioimmunoprecipitation assay buffer (Beyotime, Shanghai, China, P0013E). After being separated by SDS-PAGE and transferred to polyvinylidene difluoride membranes, the protein samples were incubated with primary and secondary antibodies at the indicated dilution. The GAPDH antibody was used as an internal control. The protein bands were detected using the electrogenerated chemiluminescence method and processed using ImageJ software (NIH, Bethesda, MD, United States).

### Statistical Analysis

All data are presented as the mean ± SD. Statistical differences were analyzed by Student’s *t*-test between two groups and one-way analysis of variance (ANOVA) with Tukey’s posthoc test for multiple comparisons. A *p*-value of <0.05 was considered statistically significant. GraphPad Prism 7.0 software was used for the statistical analysis.

## Results

### Induction of Ferroptosis in LSRA

**FIGURE 1:**
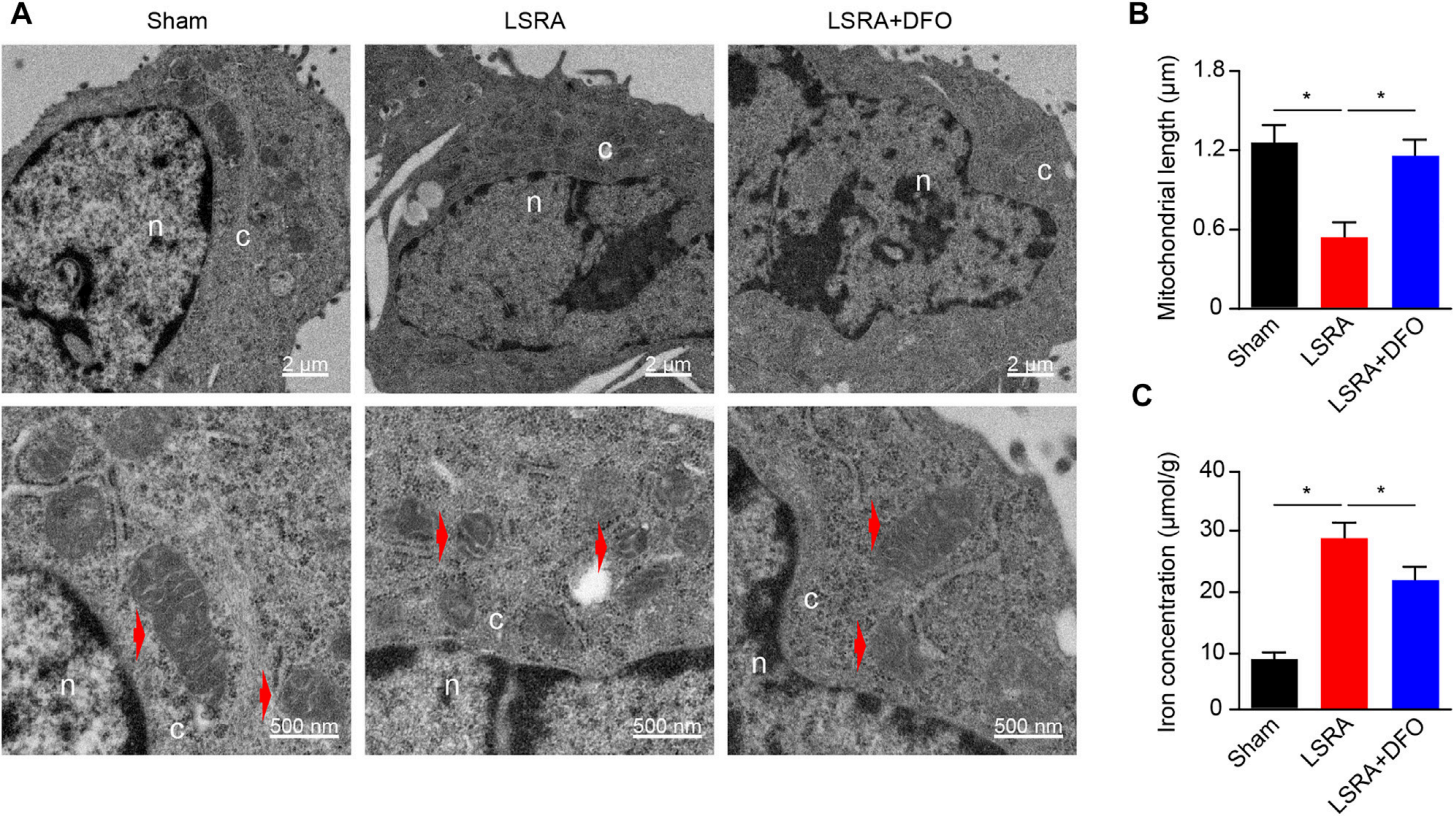
Induction of ferroptosis in spinal cord tissue after LSRA. **(A)** Transmission electron microscopy of spinal cord tissues obtained from rats in the Sham, LSRA, and LSRA + DFO groups. Scale bars: upper, 2 μm; lower, 500 nm. Red arrows indicate mitochondria. **(B)** Average mitochondrial length (along the long axis) was measured in different groups, *n* = 60 mitochondria/group. **(C)** Total iron level of spinal cord tissue was detected in different groups. *n* = 10 rats/group. Significance in **(B and C)** was calculated using the one-way ANOVA with Tukey’s posthoc test; *p < 0.05. Abbreviations: Sham, sham-operated rats; LSRA, lumbosacral spinal root avulsion; and DFO, deferoxamine.

Previous studies demonstrated that ferroptosis is characterized by the accumulation of intracellular iron and a morphological change of shrunken mitochondria (Dixon et al., 2012). To address whether ferroptosis was induced in LSRA, the ultrastructure of rat spinal cord tissues after LSRA was examined by transmission electron microscopy. We found shrunken mitochondria in the spinal cord tissues of rats of the LSRA group (Figure 1A), and compared to the Sham group, the average mitochondrial length was significantly reduced in the LSRA group (Figure 1B). However, when the rats were pretreated with DFO, a potent iron chelator and ferroptosis inhibitor, a relatively normal morphology of mitochondria was observed, and their mitochondrial length was largely retained (Figures 1A and B). Next, the iron levels of different groups were also detected (Figure 1C). The results showed that the iron level in the LSRA group was obviously increased than in the Sham group, while DFO treatment significantly decreased the iron concentration in the LSRA + DFO group compared with the LSRA group. These data demonstrated a ferroptosis-specific change in the mitochondrial morphology and iron overload in rat spinal cord tissues following LSRA, indicating that ferroptosis was induced in LSRA.

### Identification of Ferroptosis DEGs in LSRA

**FIGURE 2:**
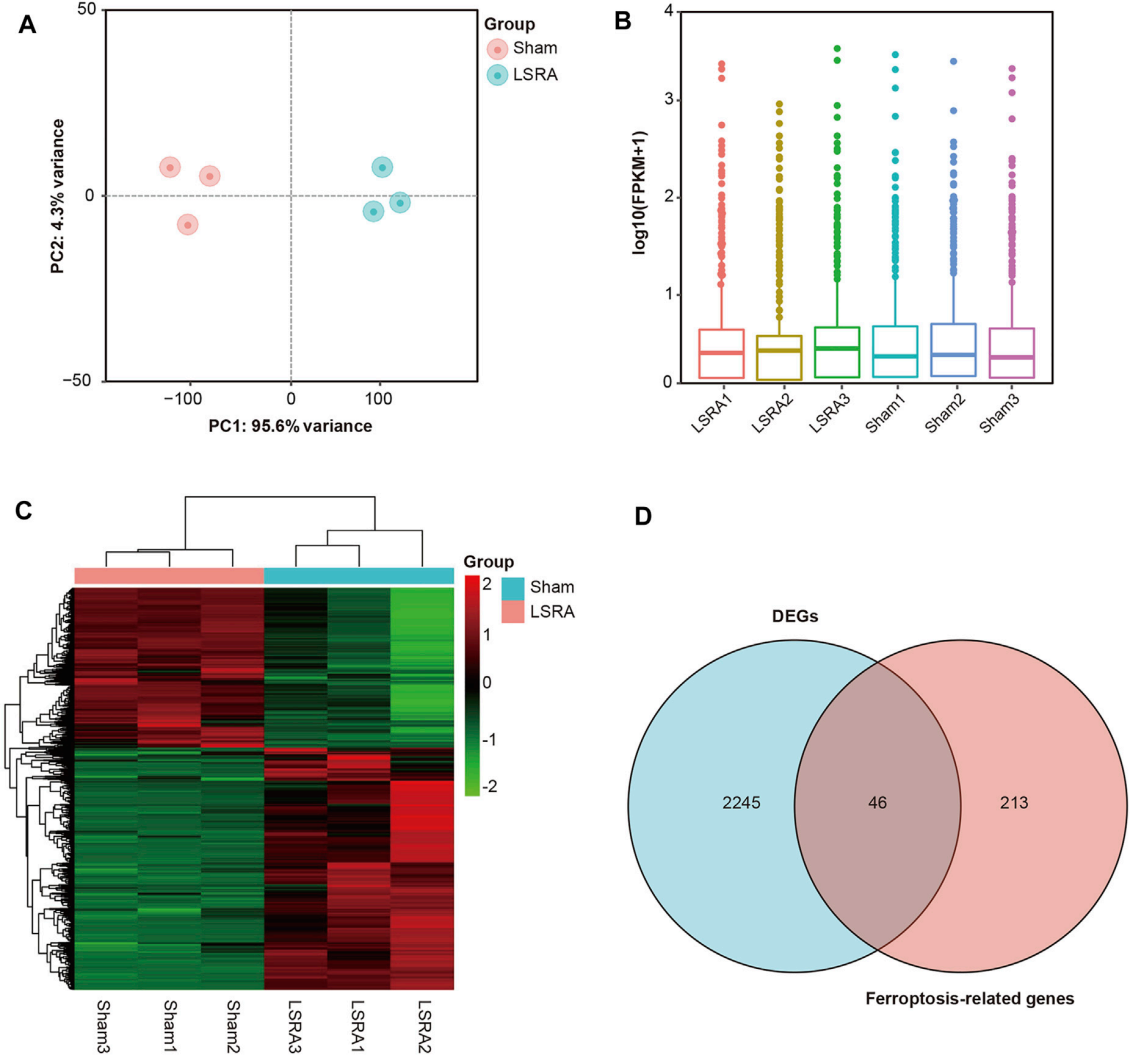
Identification of DEGs and ferroptosis DEGs in spinal cord tissue after LSRA. **(A)** Principle component analysis showing distinct clustering of the individual samples of the Sham and LSRA groups. **(B)** Box plot showing that distributions of the mRNA expression profiles in all samples were nearly the same. **(C)** The heatmap shows hierarchical clustering of DEGs in spinal cord tissue between the LSRA and sham-operated rats. **(D)** Venn diagram of ferroptosis DEGs in the spinal cord after LSRA. Abbreviations: DEGs, differentially expressed genes and LSRA, lumbosacral spinal root avulsion.

Knowing that ferroptosis was induced in LSRA, we further investigated specific ferroptosis-related genes that may involve in the pathological process of LSRA. The spinal cord tissues of the L4–L6 segments were harvested for RNA isolation and RNA sequencing. Principle component analysis showed distinct clustering of the individual samples of the Sham and LSRA groups (Figure 2A), and as indicated in the box plot, distributions of the mRNA expression profiles in all samples were nearly the same (Figure 2B), indicating the rigor and reproducibility of RNA sequencing data obtained from different samples. Then, after differential expression analyses were performed, 2,446 mRNAs were identified to be significantly differentially expressed in spinal cord tissues after LSRA, and among them, 1,470 were upregulated and 976 were downregulated. The heatmap showed hierarchical clustering of these DEGs (Figure 2C).

To identify ferroptosis DEGs in LSRA, a ferroptosis-related gene dataset (265 genes) was downloaded from the FerrDb and intersected with DEGs identified in the RNA sequencing data. As shown in Figure 2D, we found 46 ferroptosis DEGs, and among them, 40 genes were upregulated and 6 were downregulated (Supplementary Table S2). Then, based on the FerrDb online tool, these ferroptosis DEGs were further classified as ferroptosis drivers, ferroptosis suppressors, and ferroptosis markers (Supplementary Table S3).

### Functional Analysis of Ferroptosis DEGs

**FIGURE 3:**
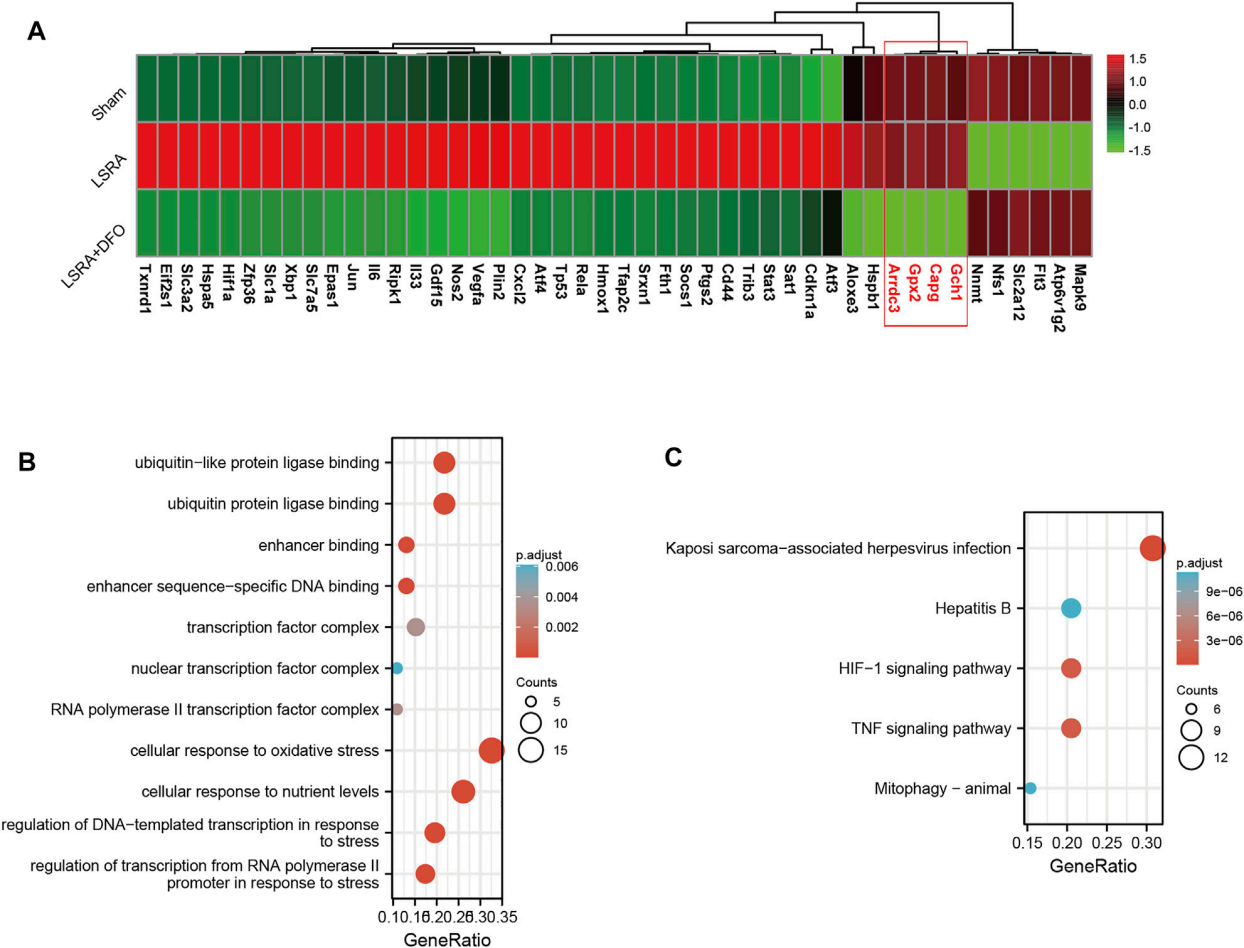
Functional analyses of ferroptosis DEGs using DAVID 6.8. **(A)** The heatmap showing qRT-PCR analysis results of 46 ferroptosis DEGs, and over 90% of the ferroptosis DEGs (except four genes: Gch1, capg, Gpx2, and Arrdc3) were confirmed to be differentially expressed following LSRA, which can also be eliminated by the DFO treatment. *n* = 10 rats/group; significance was calculated using the one-way ANOVA with Tukey’s posthoc test, *p < 0.05. GO **(B)** and KEGG pathway **(C)** enrichment analyses of ferroptosis DEGs in the spinal cord after LSRA. Size and color indicate the number of enriched genes and the degree of enrichment, respectively. Abbreviations: DEGs, differentially expressed genes; LSRA, lumbosacral spinal root avulsion; GO, gene ontology; and KEGG, Kyoto encyclopedia of genes and genomes.

Furthermore, we performed a qRT-PCR analysis for the verification of 46 ferroptosis DEGs. As shown in Figure 3A, the expression levels of over 90% of the ferroptosis DEGs (42 out of 46) were successfully verified to be consistent with the RNA-seq results, indicating the high credibility of our RNA-seq data. Besides that, we also revealed that, compared with the LSRA group, changes in the transcriptional level of these verified ferroptosis DEGs were eliminated by the DFO treatment, further suggesting their potential roles in regulating ferroptosis during the LSRA pathological process.

**FIGURE 4:**
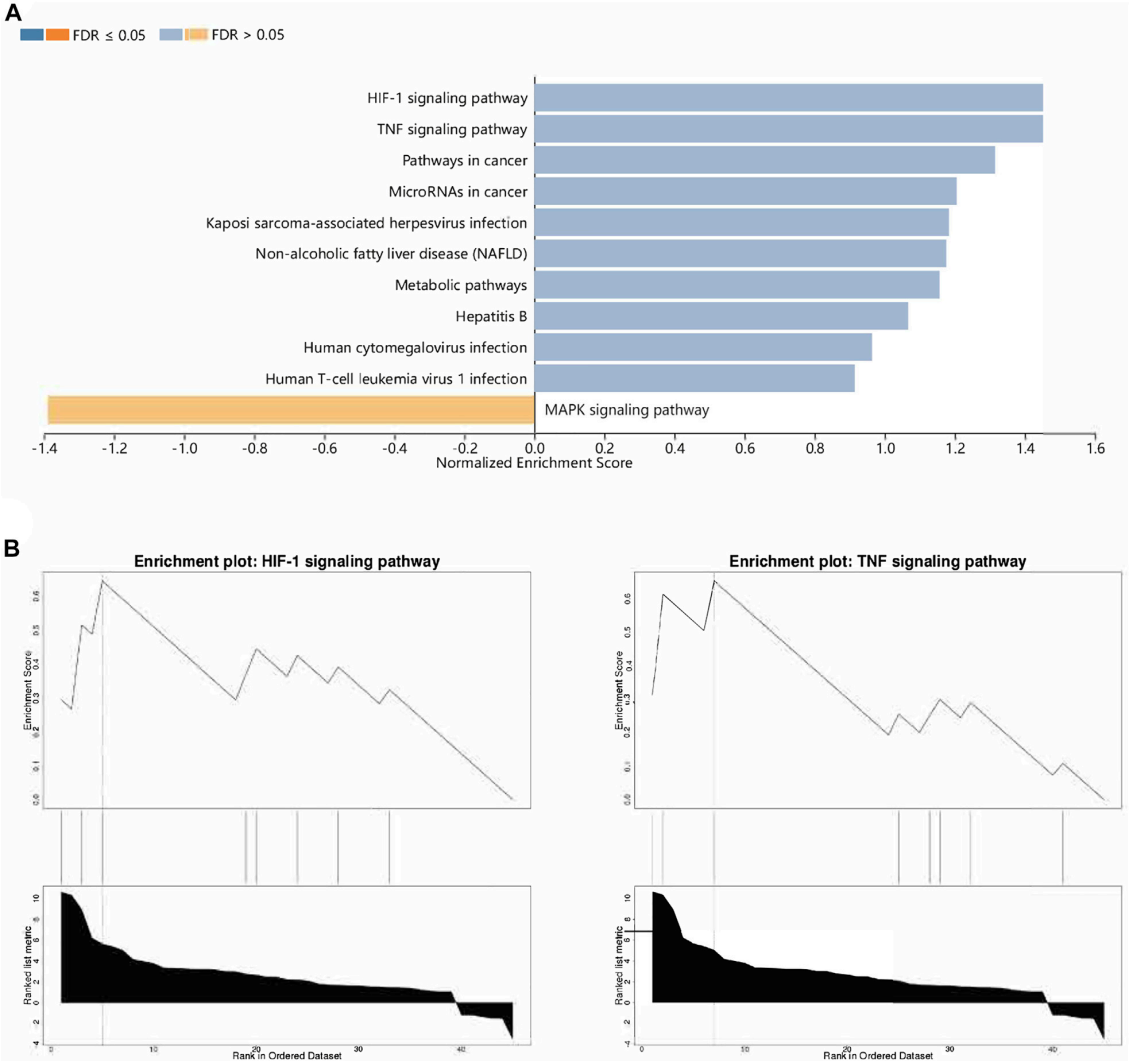
Functional analyses of ferroptosis DEGs using WebGestalt. **(A)** Enriched pathways identified by the enrichment gene dataset analyses. **(B)** Top two enriched pathways analyzed using WebGestalt. Abbreviations: DEGs, differentially expressed genes.

Then, functional analysis of the 42 verified ferroptosis DEGs identified above was performed by online tools. First, GO and KEGG pathway analyses were conducted using DAVID for the 46 ferroptosis DEGs. GO analysis revealed that the ferroptosis DEGs were remarkably enriched in biological processes, such as cellular response to oxidative stress, regulation of DNA-templated transcription in response to stress, and regulation of transcription from the RNA polymerase II promoter in response to stress (Figure 3B), and KEGG analysis found significant enrichment of the HIF-1 signaling pathway and tumor necrosis factor (TNF) signaling pathway for these ferroptosis DEGs (Figure 3C). Furthermore, GSEA conducted by WebGestalt software revealed that the HIF-1 signaling pathway, the TNF signaling pathway, and the mitogen-activated protein kinase signaling pathway were significantly activated in the gene sets (Figures 4A and B).

### Protein–Protein Interaction Analysis of Ferroptosis DEGs

**FIGURE 5:**
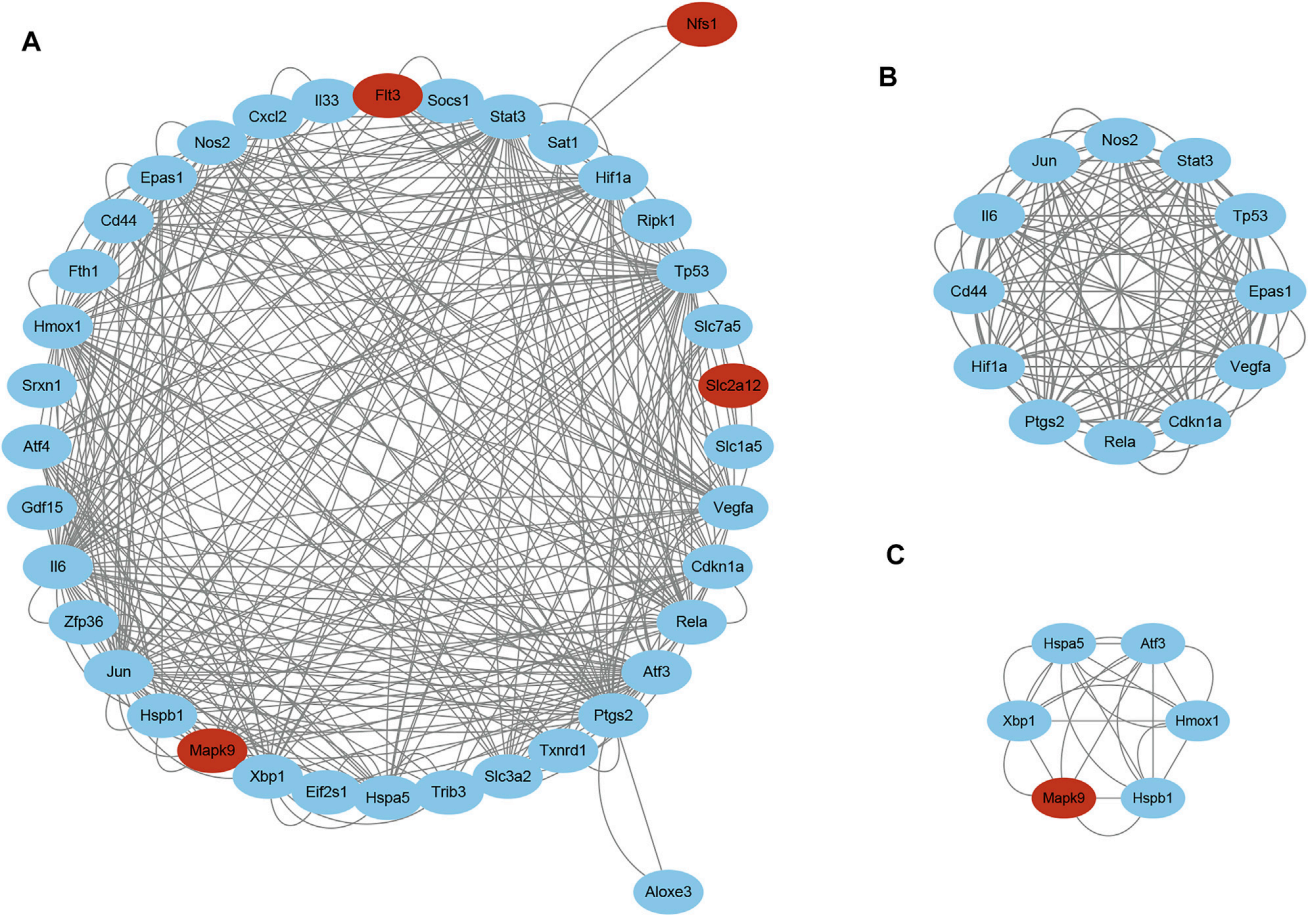
PPI network analyses of ferroptosis DEGs. **(A)** PPI network of ferroptosis DEGs visualized using Cytoscape. Red and blue nodes represent downregulated and upregulated genes, respectively. Two key modules identified using MCODE, **(B)** Cluster 1 and **(C)** Cluster 2. Abbreviations: DEGs, differentially expressed genes.

**FIGURE 6:**
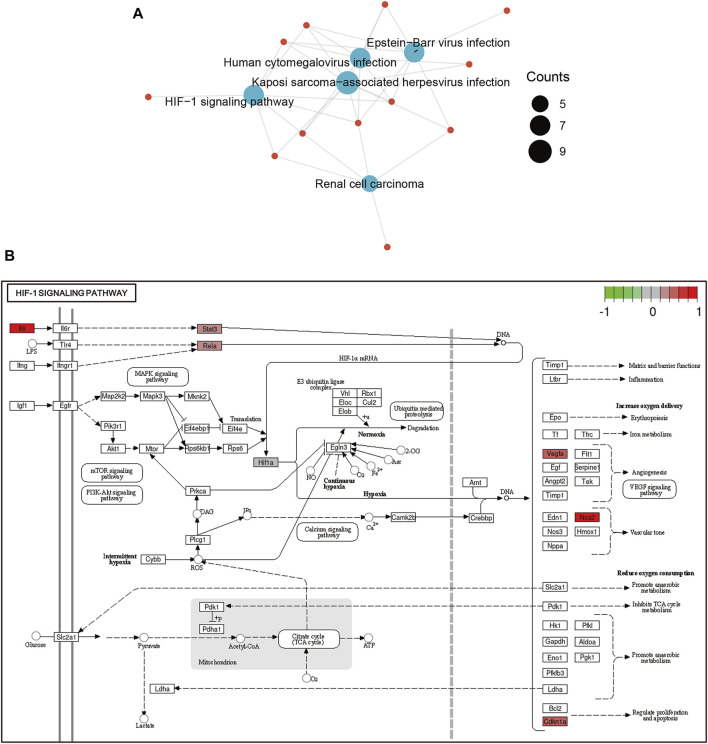
Functional analyses of ferroptosis DEGs in Cluster 1. **(A)** The HIF-1 signaling pathway was significantly enriched in Cluster 1. **(B)** Ferroptosis DEGs enriched in the HIF-1 signaling pathway were plotted using PATHVIEW. Abbreviations: DEGs, differentially expressed genes.

STRING was used to predict the PPIs of ferroptosis DEGs. Finally, 38 of the 42 genes were identified to be related to each other with the default cutoff (interaction score > 0.4). A PPI network containing 38 nodes and 366 edges was constructed using Cytoscape, in which the upregulated and downregulated genes were labeled in blue and red, respectively (Figure 5A). Furthermore, a clustering analysis of the network was performed using MCODE and three key modules were identified (Supplementary Table S4). The top two clusters contained 18 key genes, including one downregulated gene and 17 upregulated genes (Figures 5B and C). Besides that, 12 genes in Cluster 1 were uploaded for further KEGG enrichment analysis, and the results also showed that 7 of 12 genes, including Il6, Nos2, Stat3, Hif1a, Vegfa, Cdkn1a, and Rela, were significantly enriched in the HIF-1 signaling pathway (Figures 6A and B).

### Further miRNA Prediction

**FIGURE 7:**
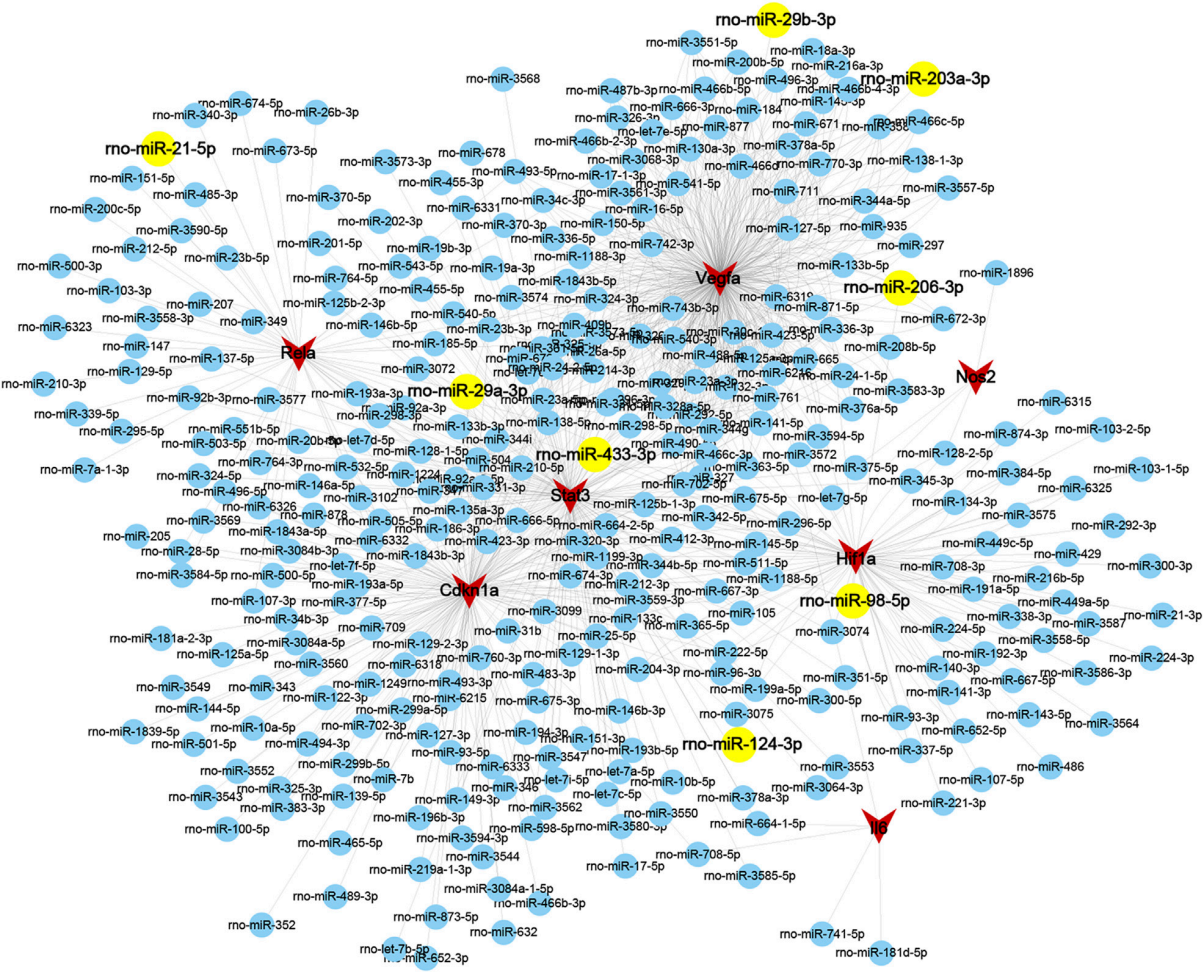
Interaction gene-miRNAs network of ferroptosis DEGs related to the HIF-1 signaling pathway in Cluster 1. Genes and miRNAs are presented as circular nodes (both in blue and yellow) and triangular nodes in red, respectively, and the miRNAs predicted by the miRTarBase database are colored in yellow. Abbreviations: DEGs, differentially expressed genes.

To further reveal the potential mechanisms of key modules in LSRA, miRWalk 2.0 was applied to predict gene–miRNA interactions of the 7 genes in Cluster 1 that were enriched in the HIF-1 signaling pathway, and 329 miRNAs were identified. To further confirm the predicted miRNAs, miRTarBase, an experimentally validated database, was used to screen the targeted miRNAs. In all, four of the 7 genes, Rela, Stat3, Hif1a, and Vegfa, were predicted to be targeted by eight miRNAs in the database, rno-miR-29a-3p, rno-miR-29b-3p, rno-miR-21-5p, rno-miR-98-5p, rno-miR-206-3p, rno-miR-433-3p, rno-miR-203a-3p, and rno-miR-124-3p (Supplementary Table S5). All of these microRNA–target interactions were visualized using Cytoscape and are shown in Figure 7.

### Verification of Key Ferroptosis DEGs

**FIGURE 8:**
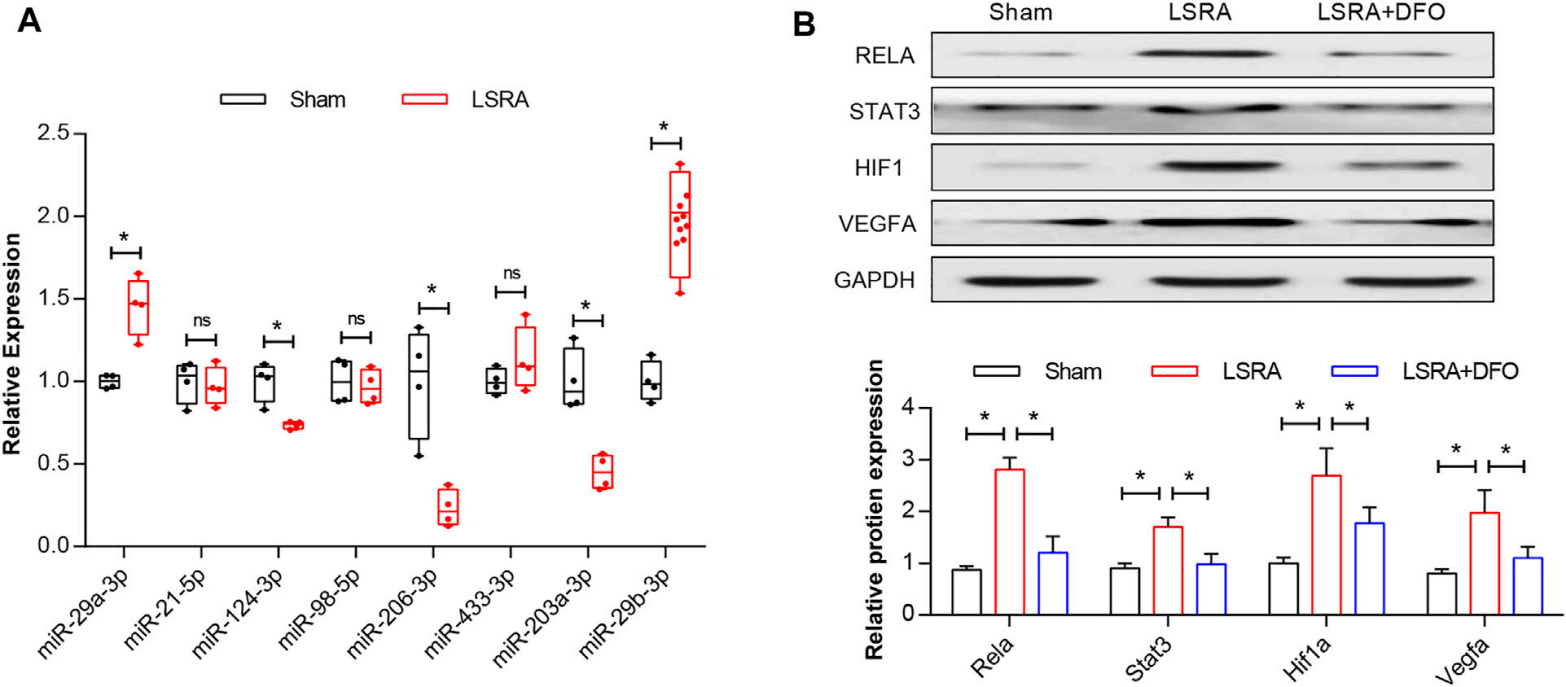
Verification of key ferroptosis DEGs. **(A)** qRT-PCR analysis of predicted miRNAs. *n* = 10 rats/group; significance was calculated using the Student’s *t*-test, *p < 0.05. **(B)** Western blot analysis of Rela, Stat3, Hif1a, and Vegfa. *n* = 10 rats/group; significance was calculated using the one-way ANOVA with Tukey’s posthoc test, *p < 0.05. Abbreviations: DEGs, differentially expressed genes; Sham, sham-operated rats; LSRA, lumbosacral spinal root avulsion; and DFO, deferoxamine.

Four genes enriched in the HIF-1 signaling pathway and related to the response to stress and their predicted miRNAs were selected for further verification using western blot and qRT-PCR analysis, respectively. As shown in Figure 8A, compared with the sham group, the results demonstrated significant upregulation of rno-miR-29a-3p and rno-miR-29b-3p and downregulation of rno-miR-206-3p, rno-miR-203a-3p, and rno-miR-124-3p in the LSRA group, while the expression levels of other miRNAs remained unchanged. Besides that, the protein levels of Rela, Stat3, Hif1a, and Vegfa were significantly upregulated in the LSRA group, while the DFO treatment significantly decreased their expression levels in the LSRA + DFO group compared with the LSRA group (Figure 8B). Given that the expression levels of miRNA and its target gene are usually negatively correlated, these data demonstrated that rno-miR-124-3p/Stat3, rno-miR-206-3p/Hif1a, and rno-miR-203a-3p/Vegfa may be involved in the pathological process of LSRA.

## Discussion

In the present study, we investigated whether ferroptosis is induced after LSRA and the key ferroptosis-related genes and their potential function in LSRA. We found that LSRA was followed by the ferroptosis-specific changes of shrunken mitochondria and increased iron accumulation that can be alleviated by ferroptosis inhibitor DFO. The transcriptional expression profile of spinal cord tissues in a rat LSRA model was revealed, and a set of key genes related to ferroptosis as well as their possible functions in the pathological process of LSRA were further investigated. Forty-six ferroptosis DEGs were obtained from the intersection of FerrDb and DEGs in LSRA, and over 90% of the ferroptosis DEGs (42 out of 46) were successfully verified to be consistent with the RNA-seq results. Functional analysis revealed a significant enrichment of these ferroptosis DEGs in the oxidative stress response, the HIF-1 signaling pathway, and the TNF signaling pathway. PPI network analysis and construction of a gene–miRNA network identified four key ferroptosis DEGs—Stat3, Hif1a, Vegfa, and Rela and further western blot analysis confirmed their upregulation after LSRA, which can be alleviated by DFO pretreatment, indicating potential roles of these modules in the pathological process of LSRA.

LSRA is a severe nerve injury that leads to devastating dysfunction in the lower extremities (Chin and Chew, 1997; Ohlsson et al., 2013). At present, there are few effective therapies because of the limited understanding of its pathogenesis. Our previous study found that the death and apoptosis index in neurons gradually increased over time after LSRA in rats (Jiang et al., 2014). Further research revealed that heat shock protein 27 is a critical regulator of LSRA by acting as a suppressor of apoptosis (Zhou et al., 2019). In addition, another canonical cell death pathway, autophagy, was also found to be activated in neurons after LSRA, and induction of autophagy greatly improved the neuronal survival rate (Zhu et al., 2016). Ferroptosis is a nonapoptotic form of cell death characterized by the accumulation of intracellular iron and morphological changes, such as shrunken mitochondria and increased membrane density (Tang et al., 2021). Numerous studies have shown that ferroptosis is involved in various human diseases, including spinal cord injury. For example, Yao and associates reported that the inhibition of ferroptosis by DFO plays a protective role in traumatic spinal cord injury (Yao et al., 2019). Ge and colleagues demonstrated that ferroptosis decreased by ferrostatin-1 markedly mitigated white matter injury after spinal cord injury (Ge et al., 2021). In this study, ferroptosis-specific changes in mitochondria morphology and iron overload were also identified in rat spinal cord tissues following LSRA, indicating that ferroptosis was induced in LSRA and might be involved in the pathological process of LSRA.

Differential gene expression is a vital feature in the development process of many diseases, and differential gene expression analyses have been commonly regarded as a very important step for scientific research. To further investigate specific ferroptosis-related genes that may involve in the pathological process of LSRA, DEGs were identified by differentially gene expression analysis following RNA sequencing and intersected with the ferroptosis dataset to obtain ferroptosis DEGs. Here, we reported the transcriptional expression profile of LSRA using a rat model for the first time and found that a large number of genes were differentially expressed in spinal cord tissues. These DEGs might be candidates for further exploration of the underlying molecular mechanisms involved in LSRA. In addition, 46 ferroptosis DEGs were obtained by intersecting the FerrDb gene dataset and DEGs identified in LSRA, and qRT-PCR analysis confirmed that over 90% of the ferroptosis DEGs were differentially expressed following LSRA, and their changes in transcriptional level were consistent with the RNA-seq results, which can also be eliminated by DFO treatment, suggesting a vital regulator of these ferroptosis DEGs in LSRA.

To reveal the potential function of ferroptosis DEGs in LSRA, different bioinformatics analysis tools were used for functional enrichment analyses. KEGG pathway analysis by DAVID and GSEA of WebGestalt identified significant enrichment of the HIF-1 signaling pathway and the TNF signaling pathway, and GO analysis using DAVID demonstrated that response to oxidative stress was markedly activated. Oxidative stress, mainly caused by excessive ROS that trigger lipid peroxidation to damage the plasma membrane and induce ferroptosis (Hassannia et al., 2019), has been proven to be closely related to peripheral and central nervous system diseases. Previous studies have shown that hypoxia can induce oxidative stress *via* overgeneration of ROS and subsequently regulate the HIF-1 signaling pathway by modulating HIF-1α transcription (Pialoux et al., 2009). In addition, a previous review demonstrated that the TNF signaling pathway also plays an important role in neurodegeneration by inducing oxidative stress (Fischer and Maier, 2015). Therefore, these findings suggest that both the HIF-1 signaling pathway and the TNF signaling pathway are closely related to ferroptosis following LSRA.

PPIs are essential biological processes for intracellular homeostasis; therefore, they have been regarded as a key issue for understanding cell physiology under normal and disease states. With the development of bioinformatics, PPI analysis has also become a promising method for identifying key modulators during the exploration of disease mechanisms. To identify key modules in ferroptosis DEGs, a PPI network was constructed and MCODE analysis was carried out. Three clusters were obtained and pathway analysis found that 7/12 genes in the cluster with the highest MCODE scores enriched in the HIF-1 signaling pathway, Il6, Nos2, Stat3, Hif1a, Vegfa, Cdkn1a, and Rela. These data further indicated that the HIF-1 signaling pathway and its related genes may be involved in LSRA and deserve further study.

MicroRNAs (miRNAs), endogenous noncoding RNAs that inhibit the translation of target mRNAs by directly binding to their 3′UTRs, have been identified as new targets for the diagnosis and treatment of disease (Bartel, 2009). In our study, seven genes related to the HIF-1 signaling pathway were selected for miRNA prediction, and four of the seven genes, Rela, Stat3, Hif1a, and Vegfa, were predicted to be targeted by eight miRNAs. Western blot analysis confirmed the upregulation of Rela, Stat3, Hif1a, and Vegfa at the transcriptional and protein levels, while DFO pretreatment significantly alleviated those effects, demonstrating the critical role that these genes might play in ferroptosis following LSRA. Moreover, the qRT-PCR analysis also confirmed the alterations of rno-miR-124-3p, rno-miR-206-3p, and rno-miR-203a-3p, which were negatively correlated with Stat3, Hif1a, and Vegfa, suggesting microRNA–target interactions between these mRNAs and miRNAs. Although none of these genes or miRNAs has been studied in LSRA, some are of great importance in nerve injury. Stat3 is an axon regeneration-associated molecule, with a report showing that upregulation of Stat3 caused by miRNA-124 may alleviate injury in motor neurons (Nagata et al., 2014). Evidence showed that autophagy induced by Sirt1/Hifα might be a new strategy for motor nerve regeneration (Romeo-Guitart et al., 2019). Previous data also demonstrated that Vegf release in human astrocytes could protect neuron-like SH-SY5Y cells from oxygen-glucose deprivation (Vincenzi et al., 2020). These data verified the reliability of our RNA sequencing and bioinformatics results and indicated the possible existence of a ferroptosis-related gene–miRNA regulation chain in the pathological process of LSRA.

## Conclusion

In conclusion, we demonstrated the induction of ferroptosis and reported the transcriptional expression profile of spinal cord tissues in a rat LSRA model for the first time and identified several key genes involved in ferroptosis. We found that 2,446 mRNAs were significantly differentially expressed in spinal cord tissues in a rat LSRA model, and among them, 46 ferroptosis DEGs and over 90% of the ferroptosis DEGs were successfully verified to be consistent with the RNA-seq results. Functional analysis revealed that these ferroptosis DEGs were enriched in the HIF-1 signaling pathway and the TNF signaling pathway. Furthermore, key genes significantly related to the HIF-1 signaling pathway (Stat3/Hif1a/Vegfa), and their associated miRNAs (rno-miR-124-3p/rno-miR-206-3p/rno-miR-203a-3p), were found to be extremely important in ferroptosis after LSRA and might be potential biomarkers in the ferroptosis pathway. Our results demonstrated the induction of ferroptosis in a rat LSRA model and identified possible regulatory roles for ferroptosis-related genes in LSRA, which may provide new insights into the pathogenesis and help to find new molecular targets for the treatment of LSRA.

## Data Availability

The original contributions presented in the study are included in the article/Supplementary Material, further inquiries can be directed to the corresponding author.
