# Supplementary material for: Identification of Potential Ferroptosis Key Genes in the Pathogenesis of Lumbosacral Spinal Root Avulsion by RNA Sequencing and Bioinformatics Analysis
Source: Front Mol Biosci. 2022 Aug 5;9:902607. doi: 10.3389/fmolb.2022.902607 (PMC9389045; doi:10.3389/fmolb.2022.902607)
Supplement: Supplementary file 1 [file Table1.DOCX]

Table S1 primers used for quantitative real-time PCR.

| Rela | Forward（5'-3'） | CGTGAGAACTGAATTCCATG |
| --- | --- | --- |
|  | Reverse（5'-3'） | GTGCAGGGTCCGAGGT |
| Stat3 | Forward（5'-3'） | CGTCAGTGCACTACAGAACT |
|  | Reverse（5'-3'） | GTGCAGGGTCCGAGGT |
| Hif1a | Forward（5'-3'） | CGTTCCTTCGATCAGTTGTC |
|  | Reverse（5'-3'） | TCAGTGGTGGCAGTGGTAGT |
| Vegfa | Forward（5'-3'） | GGACCCTGGCTTTACTGCTGTACC |
|  | Reverse（5'-3'） | TCACCGCCTTGGCTTGTCACA |
| rno-miR-29a-3p | Forward（5'-3'） | CTCAACTGGTGTCGTGGAGTCGGCAATTCAGTTGAGTAACCGAT |
|  | Reverse（5'-3'） | ACACTCCAGCTGGGTAGCACCATCTGAAAT |
| rno-miR-29b-3p | Forward（5'-3'） | ACACTCCAGCTGGGTAGCACCATTTGAAA |
|  | Reverse（5'-3'） | CTCAACTGGTGTCGTGGAGTCGGCAATTCAGTTGAGAACACTGA |
| rno-miR-21-5p | Forward（5'-3'） | ACACTCCAGCTGGGTAGCTTATCAGACTGA |
|  | Reverse（5'-3'） | CTCAACTGGTGTCGTGGAGTCGGCAATTCAGTTGAGTCAACATC |
| rno-miR-124-3p | Forward（5'-3'） | TGCGGTAAGGCACGCGGTGAATGCC |
|  | Reverse（5'-3'） | CCAGTGCAGGGTCCGAGGT |
| rno-miR-98-5p | Forward（5'-3'） | AACAATACAACTTACTACCTCA |
|  | Reverse（5'-3'） | GTGCAGGGTCCGAGGT |
| rno-miR-206-3p | Forward（5'-3'） | CGTAAAGTGCTGACAGTGC |
|  | Reverse（5'-3'） | GTGCAGGGTCCGAGGT |
| rno-miR-433-3p | Forward（5'-3'） | CGATCATGATGGGCTCCTCG |
|  | Reverse（5'-3'） | GTGCAGGGTCCGAGGT |
| rno-miR-203a-3p | Forward（5'-3'） | GTGCAGGGTCCGAGGTATT |
|  | Reverse（5'-3'） | GCCGCGTGAAATGTTTAGGACCAC |
| Gapdh | Forward（5'-3'） | GGGTCCCAGCTTAGGTTCATC |
|  | Reverse（5'-3'） | AATCCGTTCACACCGACCTT |
| U6 | Forward（5'-3'） | CTCGCTTCGGCAGCACA |
|  | Reverse（5'-3'） | AACGCTTCACGAATTTGCGT |

Table S2 Ferroptosis differentially expressed genes of Lumbosacral nerve root avulsion.

| Gene symbol | Gene title | Log2FoldChange | P-value |
| --- | --- | --- | --- |
| **Upregulated genes** | |  |  |
| Fth1 | Ferritin heavy chain 1 | 1.07748 | 1.14E-02 |
| Hspb1 | Heat shock protein family B (small) member 1 | 1.08046 | 7.57E-03 |
| Arrdc3 | Arrestin domain containing 3 | 1.12237 | 1.96E-02 |
| Eif2s1 | Eukaryotic translation initiation factor 2 subunit 1 | 1.2468 | 5.32E-03 |
| Epas1 | Endothelial PAS domain protein 1 | 1.41447 | 3.68E-03 |
| Slc3a2 | Solute carrier family 3 member 2 | 1.44957 | 1.16E-03 |
| Hif1a | Hypoxia inducible factor 1 subunit alpha | 1.47703 | 7.47E-04 |
| Jun | Jun proto-oncogene, AP-1 transcription factor subunit | 1.53042 | 3.27E-04 |
| Xbp1 | X-box binding protein 1 | 1.54855 | 3.93E-04 |
| Hspa5 | Heat shock protein family A (Hsp70) member 5 | 1.64913 | 8.97E-05 |
| Atf4 | Activating transcription factor 4 | 1.65755 | 1.05E-04 |
| Tp53 | Tumor protein p53 | 1.66443 | 1.14E-03 |
| Rela | RELA proto-oncogene, NF-kB subunit | 1.69639 | 9.09E-04 |
| Aloxe3 | Arachidonate lipoxygenase 3 | 1.71941 | 1.98E-02 |
| Txnrd1 | Thioredoxin reductase 1 | 1.83052 | 2.02E-05 |
| Ripk1 | Receptor interacting serine/threonine kinase 1 | 2.11172 | 1.30E-03 |
| Stat3 | Signal transducer and activator of transcription 3 | 2.21745 | 2.76E-07 |
| Slc7a5 | Solute carrier family 7 member 5 | 2.23839 | 1.86E-02 |
| Il33 | Interleukin 33 | 2.47496 | 2.54E-04 |
| Sat1 | Spermidine/spermine N1-acetyltransferase 1 | 2.48279 | 5.23E-03 |
| Cdkn1a | Cyclin dependent kinase inhibitor 1A | 2.67454 | 3.46E-09 |
| Vegfa | Vascular endothelial growth factor A | 2.76455 | 3.77E-03 |
| Srxn1 | Sulfiredoxin 1 | 3.00306 | 1.40E-11 |
| Gch1 | GTP cyclohydrolase 1 | 3.01339 | 5.12E-03 |
| Zfp36 | ZFP36 ring finger protein | 3.18973 | 6.15E-10 |
| Gdf15 | Growth differentiation factor 15 | 3.22459 | 6.29E-04 |
| Slc1a5 | Solute carrier family 1 member 5 | 3.2345 | 1.27E-03 |
| Capg | Capping actin protein, gelsolin like | 3.28587 | 4.84E-03 |
| Trib3 | Tribbles pseudokinase 3 | 3.32062 | 3.86E-04 |
| Atf3 | Activating transcription factor 3 | 3.35687 | 1.85E-09 |
| Socs1 | Suppressor of cytokine signaling 1 | 3.79565 | 1.76E-10 |
| Cd44 | CD44 molecule (Indian blood group) | 3.99258 | 7.95E-05 |
| Gpx2 | Glutathione peroxidase 2 | 4.17081 | 3.03E-15 |
| Ptgs2 | Prostaglandin-endoperoxide synthase 2 | 5.05458 | 2.96E-03 |
| Plin2 | Perilipin 2 | 5.41315 | 2.41E-07 |
| Hmox1 | Heme oxygenase 1 | 5.66489 | 4.95E-30 |
| Tfap2c | Transcription factor AP-2 gamma | 6.19389 | 2.84E-02 |
| Nos2 | Nitric oxide synthase 2 | 8.9394 | 7.70E-05 |
| Cxcl2 | C-X-C motif chemokine ligand 2 | 10.3439 | 8.60E-39 |
| Il6 | Interleukin 6 | 10.6419 | 3.75E-58 |
| **Downregulated genes** | | |  |
| Nnmt | Nicotinamide N-methyltransferase | -3.5243 | 1.53E-05 |
| Nfs1 | NFS1 cysteine desulfurase | -1.5349 | 5.40E-03 |
| Slc2a12 | Solute carrier family 2 member 12 | -1.51 | 1.49E-02 |
| Flt3 | Fms related tyrosine kinase 3 | -1.3587 | 6.68E-03 |
| Mapk9 | Mitogen-activated protein kinase 9 | -1.187 | 1.06E-02 |
| Atp6v1g2 | ATPase H+ transporting V1 subunit G2 | -1.1859 | 1.09E-02 |

Table S3 The ferroptosis differentially expressed genes were divided into ferroptosis driver, suppressor, and marker.

| **Driver** | **Supperssor** | **Marker** |
| --- | --- | --- |
| Aloxe3, Atf3, Epas1, Flt3, Hif1a, Hmox1, Mapk9, Sat1, Slc1a5, Socs1, Tp53 | Atf4, Cd44, Cdkn1a, Fth1, Gch1, Hif1a, Hmox1, Hspa5, Hspb1, Jun, Nfs1, Plin2, Slc3a2, Stat3, Tp53, Zfp36 | Arrdc3, Atf3, Atf4, Atp6v1g2, Capg, Cxcl2, Eif2s1, Fth1, Gdf15, Gpx2, Hmox1, Hspb1, Il33, Il6, Nnmt, Nos2, Ptgs2, Rela, Ripk1, Slc2a12, Slc3a2, Slc7a5, Srxn1, Tfap2c, Trib3, Txnrd1, Vegfa, Xbp1 |

Table S4 Molecular complex detection was used to process the data downloaded from the STRING to further mining gene clusters.

| **Cluster** | **Score (Density*#Nodes)** | **Nodes** | **Edges** | **Node IDs** |
| --- | --- | --- | --- | --- |
| 1 | 11.273 | 12 | 124 | Rela, Vegfa, Nos2, Il6, Stat3, Ptgs2, Tp53, Jun, Cdkn1a, Hif1a, Cd44, Epas1 |
| 2 | 4.8 | 6 | 24 | Hspa5, Hmox1, Atf3, Xbp1, Mapk9, Hspb1 |
| 3 | 3 | 3 | 6 | Slc2a12, Slc7a5, Slc1a5 |

Table S5 predicted miRNAs and its target genes.

| **Gene** | **miRNA in miRTarBase** |
| --- | --- |
| Rela | rno-miR-29a-3p, rno-miR-21-5p |
| Stat3 | rno-miR-124-3p, rno-miR-98-5p |
| Hif1a | rno-miR-206-3p, rno-miR-433-3p |
| Vegfa | rno-miR-203a-3p, rno-miR-29b-3p, rno-miR-29a-3p |
